# Sports Injuries in Basketball, Handball, and Volleyball Players: Systematic Review

**DOI:** 10.3390/life15040529

**Published:** 2025-03-24

**Authors:** Vladan Milić, Oliver Radenković, Ilma Čaprić, Raid Mekić, Nebojša Trajković, Omer Špirtović, Admira Koničanin, Milovan Bratić, Rifat Mujanović, Adem Preljević, Benin Murić, Izet Kahrović

**Affiliations:** 1 Department of Biomedical Sciences, State University of Novi Pazar, 36300 Novi Pazar, Serbia; oradenkovic@np.ac.rs (O.R.); icapric@np.ac.rs (I.Č.); ospirtovic@np.ac.rs (O.Š.); akonicanin@np.ac.rs (A.K.); bratic_milovan@yahoo.com (M.B.); rmujanovic@np.ac.rs (R.M.); apreljevic@np.ac.rs (A.P.); bmuric@np.ac.rs (B.M.); ikahrovic@np.ac.rs (I.K.); 2 Faculty of Sport and Physical Education, University of Niš, 18000 Niš, Serbia; nele_trajce@yahoo.com

**Keywords:** sports injuries, knee, ankle, basketball, handball, volleyball

## Abstract

Sports injuries were prevalent across various sports and resulted in temporary or permanent limitations in an athlete’s competitive performance. This research aimed to compile and analyze studies on sports injuries among basketball, handball, and volleyball players, with a particular focus on their frequency, nature, and variations based on gender and player position. A systematic search was conducted using digital databases, including PubMed, MEDLINE, ERIC, Google Scholar, and ScienceDirect, covering the period from 2015 to 2025. The search strategy involved relevant keywords and their combinations related to injuries and athletes, selecting studies that explored injury types, locations, and preventive measures. The findings indicated that lower limb injuries were the most prevalent in all three sports. Basketball players frequently experienced knee and ankle injuries, and handball players were prone to knee injuries, while volleyball players most commonly sustained knee and foot injuries. Additionally, specific risk factors contributing to these injuries were identified. Preventive interventions, such as neuromuscular training and plyometric exercises, were found to effectively reduce injury rates across these sports. The majority of studies suggested that female athletes had a higher injury incidence compared to their male counterparts. This may be attributed to several factors, including hormonal differences (such as the effect of estrogen on ligament laxity), anatomical factors (e.g., a larger Q-angle in women), and differences in training practices that could predispose female athletes to higher injury rates. Furthermore, regardless of the sport, injuries occurred more frequently during competition and tended to increase with the physical demands of the game.

## 1. Introduction

Sports such as basketball, handball, and volleyball are physically and technically demanding activities that require exceptional physical fitness, coordination, and skill proficiency [1]. These sports involve frequent jumping, abrupt directional changes, and intense interactions with opponents, leading to considerable biomechanical and physiological stress [2]. Traumatic injuries, including joint sprains, fractures, and contusions, often occur as a result of inadequate physical conditioning, improper technique, excessive strain, insufficient warm-up routines, and frequent contact with opponents, all of which significantly increase the risk of both acute and chronic injuries [3,4,5]. These factors play a critical role in injury occurrence, making them crucial areas for further investigation [6].

Preventative measures focus on appropriate warm-ups, stability exercises, correct movement techniques, and the use of suitable protective gear [7]. For instance, basketball requires dynamic actions such as sprints, jumps, and quick changes in direction [8], while handball involves high-intensity physical contact and explosive movements [9]. Conversely, volleyball is characterized by frequent jumping and landing, with repeated submaximal and maximal load jumps [10], leading to continuous strain on the lower limbs [7,11].

Research indicates that the lower extremities are the most frequently affected by injuries in these sports [12,13]. In basketball, ankle and knee injuries are the most prevalent, particularly due to the high-impact nature of the sport, which involves frequent jumps and rapid directional changes [14]. In handball, shoulder and knee injuries are the most common, largely due to frequent contact with opponents and intense ball throws [15]. Volleyball players are particularly prone to knee injuries, especially patellar tendon injuries, which result from repetitive jumps and explosive movements [7,11]. As advancements in training technology and physical demands increase, the risk of these injuries continues to rise [16]. Injury frequency also varies depending on factors such as training intensity, competition level, fatigue, and individual athletes’ physical characteristics [17,18]. The study of sports injuries includes the analysis of their mechanisms, prevention strategies, and treatment methods. Research has shown that implementing preventive programs, such as proprioceptive training, can significantly reduce the overall risk of injury [19]. Young athletes are particularly vulnerable, as they are expected to perform at a high level, often leading to an increased number of injuries [20], which escalate as the level and ranking of youth competitions rise [21]. Gender differences in injuries have also been a topic of study, with findings suggesting that female athletes are more prone to specific injuries due to anatomical, hormonal [22], and biomechanical factors. For example, female athletes are at a higher risk for knee injuries, particularly ACL tears, due to anatomical differences such as a wider Q-angle and hormonal influences [23,24].

Specific injuries, such as joint sprains [25] and adductor injuries, have been extensively examined in the literature, highlighting the importance of proper recovery for sustaining athletic performance [26,27]. Muscular injuries are particularly prevalent in handball, occurring in 44.46% of training sessions and 58% of competitive matches [28], while musculoskeletal injuries account for 67% of total cases [29]. Among basketball players, musculoskeletal injuries are highly prevalent, with a reported rate of 78.43% [30]. In volleyball, injury incidence reaches 10.7 injuries per 1000 h of play, with acute injuries most commonly affecting the ankle, while overuse injuries primarily impact the shoulder and knee [31,32]. Modern injury prevention programs, such as FIFA 11+, have been effective in reducing injury rates across various sports [33,34,35]. However, the effectiveness of these measures in preventing complex injuries like ACL tears remains limited, emphasizing the need for more research to improve prevention strategies [36,37]. This study focuses on sports that involve intensive jumping, directional changes, and dynamic interactions with opponents, offering valuable insights into injury risks and prevention strategies in team sports with significant biomechanical demands.

The aim of this study was to collect and analyze relevant data from contemporary research on sports injuries in basketball, handball, and volleyball players, with a particular focus on the incidence and specific characteristics of injuries. Furthermore, it seeks to evaluate the effectiveness of different prevention strategies, including training methods and targeted exercises, to offer practical guidance for minimizing injury risk and improving athletic performance.

## 2. Materials and Methods

### 2.1. Review Process and Methodological Approach

The literature review was conducted in accordance with the PRISMA guidelines, which define standards for transparent, systematic, and accurate reporting of all aspects of systematic reviews and meta-analyses [38]. This structured approach ensured a transparent and replicable review process.

### 2.2. Search Strategy

A systematic search was conducted, using the databases PubMed, MEDLINE, ERIC, Google Scholar, and ScienceDirect, covering studies published from 2015 to 2025. The systematic search was performed using these databases, including studies from this period. The search terms used included the following: (“sports injuries” OR “athlete injuries”) AND (“basketball” OR “handball” OR “volleyball”) AND (“prevention” OR “rehabilitation”). Additionally, manual searches were conducted by reviewing the references of relevant studies.

Furthermore, additional search terms included “knee injuries”, “ankle sprains”, “shoulder injuries”, and “overuse injuries”. These terms were applied individually or in combination to enhance search sensitivity. The reference lists of included studies were also reviewed to identify additional relevant research.

The selection process involved a thorough examination of titles and abstracts to determine potential inclusion. Only articles published in peer-reviewed journals in the English language were considered for this review.

### 2.3. Inclusion and Exclusion Criteria

The inclusion criteria for the selected studies were as follows: studies involving amateur, professional, youth, adolescent, senior, male, or female basketball, handball, and volleyball players; research focusing on injuries specific to these sports, including both acute and chronic injuries; studies comparing injuries in basketball, handball, and volleyball with injuries in other sports; and randomized and non-randomized controlled studies published in English.

The exclusion criteria included duplicate studies, conference abstracts, case reports, review articles, and studies involving individuals diagnosed with musculoskeletal disorders, cardiovascular diseases, or those who have undergone previous surgeries that limit their mobility.

### 2.4. Data Extraction and Analysis

Extracted data included the first author’s name and year of publication, participants, injury localization, and key findings.

The data focused on the incidence, localization, and patterns of injuries among athletes in basketball, handball, and volleyball. The risk of bias was assessed using the Newcastle–Ottawa Scale for observational studies [39]. The assessment was conducted by two independent reviewers, and disagreements were resolved through discussion or, when necessary, with the involvement of a third reviewer. The Newcastle–Ottawa Quality Assessment Form for Cohort Studies in basketball, volleyball, and handball (Supplementary Tables S1–S3) demonstrated good methodological quality, with scores ranging from 6 to 8. These results indicate that the included studies met key criteria related to selection, comparability, and outcome assessment, ensuring a moderate to high level of reliability. The consistent scoring across the three sports suggests that the studies maintained methodological rigor, minimizing potential biases that could affect the validity of findings in the systematic review.

### 2.5. Data Transparency

All data utilized in this review were sourced from publicly accessible studies. Comprehensive data and findings are displayed in tables that outline the prevalence and classification of injuries among basketball, handball, and volleyball players. Data sets and full-text versions of the selected studies can be provided upon request to maintain the reproducibility and transparency of the review process.

## 3. Results

**Figure 1:**
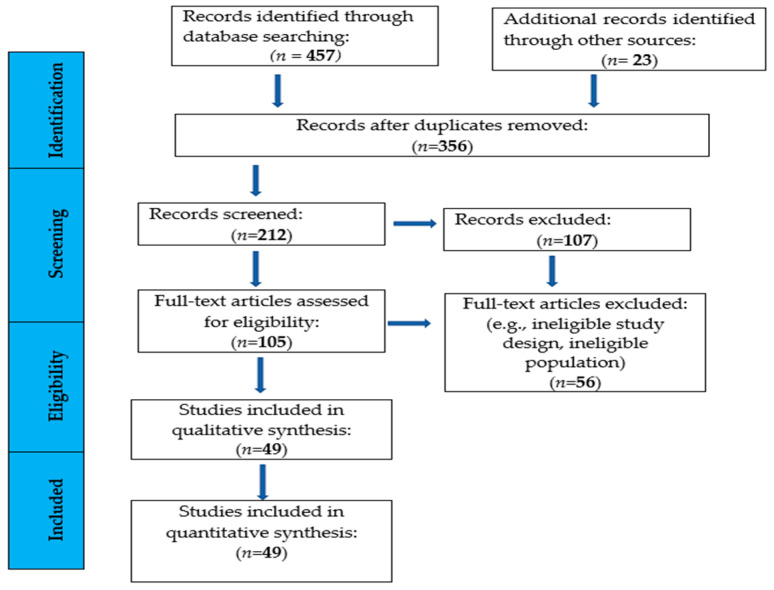
Flow chart diagram of the study selection.

A total of 457 articles were identified through database searches, with an additional 23 articles retrieved from reference lists. After removing duplicates and screening titles and abstracts, 212 studies remained. Two researchers independently evaluated these studies, and following the final screening, 49 studies were included in the systematic review. Further details on the selection process are provided in Figure 1.

### Study Characteristics

The reviewed studies encompassed participants of various ages, genders, and levels of sports activity, providing a comprehensive perspective on the frequency and types of injuries in basketball, volleyball, and handball. Special attention was given to differences in injury patterns between young and adult athletes, with younger athletes more frequently experiencing injuries related to biomechanical deficiencies, while overload and cumulative stress injuries were more prevalent among adults.

Gender-related differences were also notable, with ligament injuries being more common in women, while men were more susceptible to tendon injuries. Additionally, activity level played a significant role in injury type; professional athletes faced a higher risk of overuse injuries, whereas recreational athletes were more likely to sustain injuries due to improper technique or inadequate preparation. This analysis offers valuable insights into injury patterns across different athlete populations and serves as a foundation for developing targeted prevention programs tailored to their specific needs.

## 4. Discussion

Sports injuries are a common occurrence in team sports, with their prevalence depending on the specific demands of each discipline. The main finding of this systematic review was the analysis of injury prevalence and types among basketball players, volleyball players, and handball players, with data presented in tables providing a deeper understanding of injury distribution across these sports.

According to the data in Table 1, basketball players are most frequently exposed to lower extremity injuries, particularly ankle sprains, which can be explained by sudden direction changes, jumps, and physical contact with opponents. Table 2 shows that in volleyball, knee and finger injuries dominate, which is associated with the biomechanical load during jumps and landings. On the other hand, Table 3 indicates that handball players are most prone to shoulder and hand injuries, mainly due to intense physical contact and powerful shots.

A comprehensive analysis indicates that lower extremity injuries, particularly those involving the ankle and knee, are most common across all three sports, regardless of gender or player position. The most frequently reported injuries include ankle sprains, ligament strains, and injuries resulting from intense physical contact and jumping. Additionally, injuries are more likely to occur during competitions than during training, indicating the increased physical load and effort required during matches.

From a clinical perspective, the findings of this study provide valuable insights for therapists and injury prevention specialists, enabling them to develop targeted rehabilitation programs tailored to the specific injury patterns in each sport. Furthermore, the research holds significant importance in the field of biomechanics, as it helps identify key injury mechanisms and contributes to the development of preventive strategies aimed at reducing injury frequency, particularly those related to jumps, landings, and physical contact.

However, the interpretation of these results should take certain limitations into account. The analyzed studies did not differentiate between injury rates among professional and amateur athletes, which may impact the generalization of the findings. Additionally, comparing injury incidence during the regular season and playoff phases could provide a deeper insight into how different phases of competition affect injury risk. These points highlight the need for future research that could contribute to more precise preventive strategies and tailored rehabilitation programs based on the specific demands of each sport.

### 4.1. Basketball Injuries

Basketball is a high-intensity sport that involves fast movements, explosive actions, and frequent physical contact, which increases the risk of injuries [82]. Understanding the frequency, localization, injury mechanisms, and the effectiveness of preventive measures is crucial for preserving the health of basketball players and optimizing their performance.

#### 4.1.1. Localization and Frequency of Injuries by Gender in Basketball 

Research clearly emphasizes gender differences in sports injuries. Women experience significantly higher rates of anterior cruciate ligament (ACL) injuries than men (IRR = 2.18), with 69% of these injuries occurring without direct contact [82]. Among young athletes, girls most frequently sustain injuries to the ankle (49%) and knee (19%), whereas boys most commonly injure their ankle (46%) and thigh (10%) [45]. In elite male basketball players, the most prevalent injuries include muscle strains (21.2%) and ankle sprains (11.9%). In contrast, elite female basketball players experience a higher proportion of lower extremity injuries, accounting for 29.03% of all reported cases. Similarly, in NBA players, injuries to the lower extremities, particularly the knee and ankle, make up the largest percentage of injuries. The highest injury rates are observed in players who spend more than 30 min per game on the court [26,44,49].

#### 4.1.2. Injuries by Position on the Court

A player’s position on the court plays a significant role in determining the frequency and type of injuries they sustain. The highest incidence of injuries (34%) occurs in the key area, where physical contact and explosive movements are most intense. Injuries are more likely to happen during offensive plays (69%) than defensive actions (31%), with 41% occurring in the offensive half and 25% in the defensive half of the court [46,51]. Furthermore, different playing positions carry distinct injury risks. Centers and power forwards, due to frequent jumping and physical contact in the post, are most prone to ankle and knee injuries. On the other hand, guards, who rely on rapid directional changes and explosive sprints, are more susceptible to thigh muscle and tendon injuries [46,51].

#### 4.1.3. Most Common Injuries and Injury Mechanisms in Basketball 

The ankle stands out as the most commonly injured area in basketball [47,48] with sprains accounting for between 40% and 50% of all injuries in this region [26]. Among young basketball players, the incidence of ankle injuries is 21.9% [46], while in professional players, the rate is slightly lower at 26.9% [50]. In veteran basketball players, the prevalence of ankle injuries is reported at 21% [41]. Knee injuries are also highly prevalent, with some studies identifying them as the most common, accounting for 45% of all injuries [41]. Among these, meniscus injuries make up 21.2%, while ligament tears account for 15% [49]. Lower back injuries, often of a chronic nature, have been recorded in 53 athletes, representing 11.0% of the total sample [26]. Additionally, specific injuries to the pelvis and lower back affect 15.5% of basketball players [50]. A significant portion of basketball injuries occurs without physical contact or due to overuse. Contact injuries typically arise in high-intensity situations, such as battling for possession, whereas non-contact injuries are commonly linked to rapid directional changes, improper landings, and insufficient warm-ups. Notably, 60% of ACL injuries occur in non-contact scenarios [45]. Overuse injuries account for 20.3% of ankle injuries and 14.3% of knee injuries [41].

#### 4.1.4. Effectiveness of Preventive Measures in Basketball 

Preventive strategies play a crucial role in reducing injury rates in basketball. Proprioceptive training [25], which focuses on balance control and stability, has been shown to decrease the incidence of ankle injuries by 81% and knee injuries by 64.5%.

Targeted exercise programs [43] aimed at strengthening the lower extremity muscles, particularly in young athletes, can significantly lower the risk of ligament and joint injuries. Additionally, incorporating proper warm-ups and gradually increasing training intensity can reduce injury occurrence to 2.72 injuries per 1000 h of activity [48].

Different approaches to injury prevention in basketball include the following:

Proprioceptive training and balance exercises—Programs based on proprioceptive training have shown a significant reduction in injury incidence. The study [25] found that balance training reduced the frequency of ankle injuries by 81% and knee injuries by 64.5% in professional basketball players;

Lower extremity muscle strengthening programs—Exercises focusing on strengthening the thigh muscles (quadriceps, hamstrings) and ankle stabilizer muscles have proven particularly beneficial [43];

Basketball-specific preventive programs—Programs like FIFA 11+, originally developed for soccer, have shown a reduction in injuries in sports with similar biomechanical loads, including basketball (Mateos Conde et al., 2022 [47]);

Corrective warm-up programs—The study by Minghelli et al. (2022 [48] demonstrated that the implementation of targeted warm-up exercises reduced injury frequency to 2.72 injuries per 1000 training hours.

Among professional players, injuries such as Achilles tendon ruptures can have lasting effects on performance and career longevity, often leading to reduced abilities upon return to play [42]. In the NBA, adductor injuries have a 91% recovery rate [27], though more severe tears result in prolonged recovery times and an increased risk of re-injury.

### 4.2. Volleyball Injuries

Volleyball is a high-intensity sport that demands precise and rapid movements, frequent jumping and landing, and powerful ball strikes, all of which contribute to an increased risk of injury. Data from the International Volleyball Federation indicate that volleyball-related injuries make up a substantial proportion of total sports injuries, with the lower extremities and shoulders being the most commonly affected areas. This discussion explores injury prevalence based on gender and playing position, identifies the most frequent types of injuries, and examines the effectiveness of preventive measures through a comprehensive analysis of the existing literature.

#### 4.2.1. Localization and Frequency of Injuries by Gender in Volleyball 

Biomechanical differences between genders play a significant role in determining injury frequency and types. Women are more prone to knee and ankle injuries [66], largely due to anatomical factors such as a greater Q-angle and reduced muscular joint stability. Similar findings have been reported in time-loss injuries [60] where the injury rate among women was 2.62 per 1000 athlete-exposure hours, compared to 1.75 for men. Men, on the other hand, experience musculoskeletal injuries more frequently, particularly shoulder injuries caused by the high-impact nature of spiking. The majority of these injuries (85.33%) are classified as mild [64]. A study analyzing 98 female and 301 male athletes over two league seasons [58] found that 45% of all injuries involved the musculoskeletal system, with men experiencing a significantly higher injury rate (56%) compared to women (26%). This discrepancy may be attributed to differences in training intensity, physical attributes, and variations in training load strategies between genders.

However, gender-based injury rates are not consistent across all studies. For example, [62] reported that injuries among female athletes accounted for 77.6%, while the injury rate among male athletes was 22.4%. These contrasting findings highlight the importance of gender-specific injury prevention programs. Meanwhile, some studies have found no substantial differences in injury occurrence between men and women [31].

#### 4.2.2. Injuries by Playing Position on the Court

Different positions on the court impose specific biomechanical demands that increase the risk of injuries. Based on the analyzed injury incidence [31], injuries among middle blockers are more frequent compared to liberos, which may be a consequence of the biomechanical loads specific to their position. Lower back injuries, although less common (8.9%), highlight the need for preventive programs focused on strengthening core muscles and improving movement techniques to reduce the risk of chronic issues. The most frequently occurring injuries were located in anatomical regions such as the ankle, knee, and shoulder, with sprains, tendinopathies, and strains being the most prevalent types. These injuries primarily occurred during blocking and attacking actions at various points throughout the season [57].

#### 4.2.3. Most Common Injuries and Injury Mechanisms in Volleyball 

Ankle injuries are the most common in volleyball [54], accounting for 25.9% of all injuries [31], followed by knee injuries at 15.2%, and finger/thumb injuries at 10.7%. In contrast, knee injuries were recorded in 58% of players [61] and were highlighted as the most prevalent [58], particularly anterior cruciate ligament (ACL) injuries at 15.2% [52]. A study analyzing the frequency and types of injuries among athletes [56] during the 2013/2014 season on a sample of 94 athletes reported similar findings, where the most common injuries were knee injuries (25.9%) and ankle injuries (8.4%). Lower extremities were injured more frequently (40.8%) than upper extremities (34.3%) [13]. The lower back, along with the knee and ankle, is among the most common injuries in volleyball, occurring in 13.3% of cases [56], less than 10% [31], while lower back and shoulder injuries were recorded in 27% of players [61]. Although not insignificant in number, spinal injuries are usually mild [53].

Traumatic injuries account for 17%, while 83% are due to overuse [63]. In addition to acute injuries, such as sprains and ligament strains, chronic musculoskeletal injuries are also common, especially among professional players.

The injury incidence among high school and collegiate volleyball players, monitored over a four-year study [55], indicates that the injury rate in college volleyball was 3.3 times higher than in high school. Knee injuries were significantly more frequent among college players, with a high proportion of pressure-related injuries (38.1%), while acute anterior cruciate ligament (ACL) injuries (9.3%) were more dominant in high school players. Additionally, classification by diagnosis shows that patellar tendinosis (19.3%) was the most common condition among college players. Shoulder injuries were also more frequent among college athletes, primarily due to the forces of impact or compression acting on the joint during intense game movements (37.8% in college players and 39.1% in high school players).

At least one musculoskeletal injury [59] is reported in 67% of young volleyball players aged 13–18 years, with the most common injuries being ankle (40.6%), fingers (36.6%), knee (21.2%), and shoulder (15.5%) injuries.The results showed that injuries were more frequent among advanced volleyball players (73%) compared to beginners (62%), which can be associated with a higher training volume among advanced athletes (490 h vs. 302.3 h for beginners). Interestingly, beginners and intermediate-level players had a higher risk of elbow injuries compared to advanced players, which may indicate insufficient technical preparedness and adaptation to training loads.

#### 4.2.4. Effectiveness of Preventive Measures in Volleyball 

Insufficiently healed previous injuries, lack of adequate rest, and exhaustion are highlighted as the main causes of injuries [54]. Preventive measures, such as muscle-strengthening and joint-stabilization programs, have proven highly effective in reducing the risk of injuries. Programs focused on proper landing techniques and flexibility [66] significantly lower the risk of knee injuries.The inclusion of targeted exercise programs, consisting of stretching, strengthening muscles and ligaments around joints, and improving spinal and core stability [58], is a key part of preventive strategies to reduce the incidence of musculoskeletal injuries.

In volleyball, several key factors play an important role in injury prevention:

Training proper landing techniques—The study [61] emphasizes the importance of biomechanical correction of landing techniques, which significantly reduces the risk of anterior cruciate ligament (ACL) injuries in female volleyball players;

Proprioceptive training and joint stabilization—The study [56] showed that proprioceptive training reduces knee injuries by 25.9% and ankle injuries by 8.4%;

Strengthening lower extremity muscles and flexibility—Programs aimed at strengthening the quadriceps and calf muscles (particularly eccentric quadriceps strengthening exercises) have significantly reduced patellar tendon injuries [55];

Reduction in overtraining—Higher training loads increase the risk of injury. The study [63] highlights that reducing sudden changes in training load decreases knee injuries with *p* = 0.003.

These findings emphasize the importance of timely and adequate treatment in the recovery process, as well as the need for consistent prevention to reduce the frequency of injuries among athletes. However, failure to implement preventive measures consistently [65] increases the injury rate.

Injury treatment for volleyball players includes both conventional and alternative approaches, with acupuncture being the most commonly utilized method (40.4%). In contrast, Chuna therapy (16.0%) and physical therapy (15.2%) were used less frequently [56].

### 4.3. Handball Injuries

Handball is an intense sport that combines speed, agility, strength, and contact, making it one of the sports with a high injury rate. Injuries in handball can result from contact with opponents, improper movements, overuse, or poor preparation. The most common injuries include knee, ankle, shoulder, and head injuries. These injuries significantly affect athletes’ performance and their ability to return to play.

#### 4.3.1. Localization and Frequency of Injuries by Gender in Handball 

The analysis of studies has shown a significant difference in injury frequency between men and women. Women are 1.5 times more likely to sustain an injury compared to men, which has also been confirmed through position-specific data, where line players were at a higher risk of injury compared to wing players [3].The injury incidence rate for women is 6.21 per 1000 h, while for men, it is 4.39 per 1000 h [75]. The injury incidence during matches was 27.7 injuries per 1000 h for women, while for men it was 10.6 injuries per 1000 h. This difference was statistically significant (χ^2^ = 7.32, *p* < 0.001, CI 95%: 1.12–1.56) [75,76]. Possible causes for this difference include the following: Biomechanical factors—women have a greater Q-angle, which may increase stress on the knees and predispose them to injuries such as ACL ruptures [24]. Hormonal factors—the influence of estrogen on ligamentous stability may contribute to a higher risk of injuries [22]. Differences in training and preparation—some studies suggest that women are less likely to participate in proprioceptive training programs and preventive exercises, which may increase the incidence of lower extremity injuries [25]. In addition to these differences, more drastic gender disparities in injury rates were found [76], where women had nearly three times higher chances of getting injured per 1000 h of match play and almost twice the risk of injury per 1000 h of training compared to men. Among adolescents, the injury rate differences are similar to those found in adult athletes, where women are twice as likely to sustain an injury as men [77]. In younger age categories, higher injury rates were recorded in boys than in girls, with knee and head injuries being the most common in both genders [73]. Biomechanical differences between genders, including muscle strength and joint stability, play a significant role in variations in injury risk [72].

#### 4.3.2. Injuries by Playing Position

Player injuries, in addition to the usual classifications based on location, type, and mechanism of occurrence, have also been recorded and analyzed according to playing position [78,79,83]. Line players are the most frequently exposed to injuries, particularly in the ankle, thigh, and knee regions [83]. Backcourt and wing players have the highest absence rates per 1000 h of play, although no statistically significant differences in injury incidence among positions were recorded [79]. Comparing different positions, studies have shown that line players have a higher injury rate than wing players, while backs are more prone to knee and cartilage injuries [3,71]. Conversely, some research indicates that the most common injuries occur among left and right backs, accounting for 41% of all injuries [76]. Intense physical contact and frequent changes in direction further increase the risk of injuries among players in specific positions [73].

#### 4.3.3. Most Common Injuries and Injury Mechanisms in Handball 

The knee and ankle have been identified as the most commonly injured body parts in handball players [3,70,72,74,76,80,81,83]. Overuse injuries, such as tendinopathies and lower back issues, are also frequently reported [68,69]. Knee and ligament injuries have the highest incidence of long-term consequences, including permanent medical impairment [72]. Recurrent injuries account for nearly half of all injuries [80]. Head and facial injuries, though less common, pose a serious risk due to potential consequences such as concussions [73]. Shoulder injuries are often associated with overuse during shooting, which is a trend observed across all age groups [67].

#### 4.3.4. Effectiveness of Preventive Measures in Handball 

Research indicates that injury prevention programs emphasizing knee stability, balance enhancement, and load management play a critical role in minimizing injury risks [80]. Reducing excessive load [67] is particularly beneficial for athletes with limited external shoulder rotation or scapular dyskinesis. Additionally, knee stabilization programs have been shown to significantly decrease the likelihood of severe injuries among handball players of all age groups [72]. Preventative strategies that incorporate muscle strengthening and flexibility training are especially effective in lowering the occurrence of overuse injuries [68,69].

A structured approach to load monitoring and individualized training—tailored to factors such as gender, playing position, and age—is essential for maintaining overall health, minimizing injury risk, and optimizing athletic performance. This method allows for personalized training adaptations that cater to each athlete’s specific requirements, promoting both physical development and peak performance in sports like basketball, volleyball, and handball. Regular assessment and adjustments to training regimens contribute to faster recovery, improved game efficiency, and the long-term sustainability of an athlete’s career.

### 4.4. Review of Studies with Different Research Designs

Our results are consistent with previous studies that analyzed the epidemiology of sports injuries in basketball, handball, and volleyball. A prospective epidemiological study that [84] conducted on basketball injuries over the course of one competitive season identified ankle sprains and knee injuries as the most common. These findings were further confirmed by the work of [85], who thoroughly analyzed the mechanisms and frequency of knee injuries in basketball, with a particular focus on anterior cruciate ligament (ACL) ruptures as one of the most common injuries among high-level competitive players. Given that our analysis indicates a high prevalence of knee injuries in basketball players, particularly female athletes, our findings align with the aforementioned studies.

Regarding volleyball, the results of our study confirm the findings of [86] who identified the ankle, knee, and shoulder as the anatomical regions most commonly affected by sports injuries in this sport. These data are further supported by the research of [87], who pointed to the significant frequency of patellar and knee ligament overuse injuries in volleyball players, which aligns with our findings showing a high incidence of knee injuries, especially among players in positions that require frequent vertical loads during jumps and landings.

Similarly, in handball, our findings suggest a higher incidence of knee injuries compared to ankle injuries, which partially differs from earlier epidemiological studies. In their study on the etiology of handball injuries, ankle sprains were identified as the dominant injury by [88]. Similar results were obtained in the research by [89] who analyzed the epidemiology of injuries in young handball players. The possible discrepancy between these studies and our findings may be explained by differences in the sample population, methodological approaches, and changes in the physical demands of the sport over time. Specifically, [88] analyzed a population of recreational and youth athletes, whereas our review includes data encompassing professional athletes, who experience higher biomechanical loads, which may contribute to the increased incidence of knee injuries compared to ankle injuries.

In this context, the study by [90], which analyzed the prevalence of sports injuries in adolescents in basketball, handball, and football, identified knee and ankle injuries as the most common injuries in these sports. These findings align with our results, confirming that lower extremity injuries are dominant in sports that involve sudden changes in direction, jumps, and intense contact situations. Considering all these findings, it is clear that lower extremity injuries, particularly knee injuries, constitute a significant portion of injuries in sports such as basketball, volleyball, and handball, highlighting the need for specific biomechanical and training approaches to reduce the risk of these injuries.

In addition to analyzing the epidemiology of sports injuries, it is essential to critically assess the effectiveness of existing preventive strategies. While there is strong evidence supporting the effectiveness of proprioceptive training, muscle strengthening programs, and joint stabilization techniques in reducing the risk of injuries, most available studies rely on cohort studies or retrospective analyses. The absence of randomized controlled trials (RCTs), which are considered the gold standard in research, complicates drawing definitive conclusions about the long-term effectiveness of these interventions. The study [43] reported a reduction in injury frequency following the implementation of a preventive program, but due to the lack of an RCT design, the reliability of the results remains limited. Similar limitations were observed in the studies [55] [74], where the effects of preventive programs were assessed without a clear control group. Therefore, further research with more rigorous methodologies is necessary to confirm the long-term effectiveness of preventive programs across different sports.

## 5. Conclusions

This systematic review highlights the high prevalence of injuries in basketball, volleyball, and handball, with a particular focus on lower extremity injuries, especially those affecting the ankle and knee, which are the most common in all the analyzed sports. Regardless of the discipline, injuries occur more frequently during competition than during training, reflecting the increased physical demands of competitive play.

The findings also indicate significant variations in injury frequency according to playing positions, with basketball guards, volleyball middle blockers, and handball players in the backcourt being at the highest risk. Additionally, gender differences in injuries are also evident, with women particularly prone to knee and shoulder injuries, while male athletes are more susceptible to injuries caused by contact and physical exertion during matches.

Injury rates also vary by age and competition demands. Although the injury frequency among adults and adolescents is similar, it remains higher compared to younger athletes, with the frequency increasing as competition intensity rises. Biomechanical factors such as jumping, landing, and contact with opponents play a key role in injury occurrence across all three sports.

While various injury prevention programs have been implemented—including proprioceptive training, muscle strengthening programs, and corrective warm-up routines—the long-term effectiveness of these interventions remains insufficiently studied. Most research measures injury incidence over a single season, while long-term effects of preventive measures are largely unexplored. Additionally, there are limited data on athlete adherence to these programs, meaning it is unclear to what extent athletes con-sistently perform the recommended exercises and whether they continue with preventive measures after the study period. Furthermore, sport-specific biomechanical differences are not always thoroughly analyzed; for instance, landing-related injuries dominate in basketball and volleyball, whereas contact and throwing-related injuries are more prevalent in handball.

One of the major limitations of this systematic review is the lack of randomized controlled trials (RCTs), which represent the gold standard for evaluating the effectiveness of injury prevention strategies. Most analyzed studies are observational, making it difficult to establish a direct cause-and-effect relationship between the implementation of preventive programs and the reduction in injury incidence. Future studies should focus on well-designed RCTs to provide a more objective evaluation of preventive strategies in basketball, handball, and volleyball.

Future research should also compare injury patterns between professional and amateur athletes, as well as examine specific phases of competition that may influence injury rates. Developing sport- and position-specific preventive programs, along with rehabilitation strategies tailored to different types of injuries, could significantly reduce injury prevalence and improve athlete recovery times.

These findings emphasize the need for better load management and more effective rehabilitation strategies to reduce the recurrence of injuries and enhance overall athlete well-being. Additionally, the implementation of long-term injury prevention monitoring and RCT-based studies would contribute to more scientifically grounded recommendations, ensuring the sustainability of athletes’ careers and their long-term sports participation.

## Figures and Tables

**Table 1:** Prevalence of sport injuries in basketball.

First Author and Year	Participants	Localization	Key Findings
Leppänen et al., (2015) [40]	N = 401	Knee	The incidence of overuse injuries was 1.0 per 1000 h. A total of 66% of basketball-related injuries involved the lower extremities, with knee injuries being the most common (45%).
Moreira, et al., (2016) [41]	N = 210 veteran basketball players	Knee and Ankle	Quality of life was linked to the level of physical activity and sports injuries. Lower extremity injuries included the following: knee (35%) and ankle (21%).
Minhas et al., (2016) [42]	N = 129 NBA players	Hand/Wrist, Knee, and Achilles Tendon	Return-to-play (RTP) rates included the following: hand/wrist fractures—98.1% and Achilles tendon injuries—70.8%. Achilles tendon injuries negatively impacted career length and performance post-recovery. Knee surgical interventions had a negative impact on performance after recovery.
Riva et al., (2016) [25]	N = 55 professional male basketball players	Ankle and Knee	Proprioceptive training, particularly balance control in a standing stance, significantly improved stability and reduced injury incidence. Injury reduction rates included the following: ankle (81%); lower back (77.8%); and knee sprains (64.5%).
Pasanen et al., (2017) [43]	N = 201 adolescent basketball players	Ankle, Back, Thigh, and Hand	Injury incidence rates included the following: 1.51 per 1000 h during training and 34.47 per 1000 matches. Most commonly injured body parts in girls included the following: ankle (49%); knee (19%); and finger (7%). Most commonly injured body parts in boys included the following: ankle (46%); knee (11%); and thigh (10%). Joint and ligament injuries were the most frequent types of injuries in both girls (69%) and boys (66%). The number of recurrent ankle sprains was 19 in girls and 16 in boys.
Garbenytė-Apolinskienė et al., (2019) [44]	N = 358 elite female basketball players	Head and Lower Back, Upper Extremities, and Lower Extremities	Out of 358 athletes, 43% experienced at least one injury, pain, or illness, indicating a high prevalence of health issues. Injury distribution (34.84% of total cases) included the following: head and lower back: 5 cases (3.23%); upper extremities: 4 cases (2.58%); and lower extremities: 45 cases (29.03%).
Anderson et al., (2019) [45]	Male and female athletes (basketball and soccer)	Knee	Between 2004 and 2016, a total of 529 ACL injuries were reported, with 320 (60%) being non-contact injuries. Women accounted for 67% of all ACL injuries and 69% of non-contact cases. The injury rate was significantly higher in women than in men (IRR = 2.18; *p* < 0.0001). A total of 264 injuries (50%) were recorded, with 172 being non-contact (54%). No significant interaction was found between gender and sport type.
Rodas et al., (2019) [26]	Professional male basketball players	Muscle Strains, Ankle, Lower back, and Knee	A total of 463 injuries were recorded: muscle strains: 98 cases (21.2%), TL rate of 58.2%, and IR = 1.3/1000 h (95% CI: 1.0–1.7) and ankle sprains: 55 cases (11.9%), TL rate of 47.3%, and IR = 0.6/1000 h (95% CI: 0.4–0.9). Tendinopathies and fasciitis were recorded: 97 cases (21.0%); ligament sprains in the foot: 54 cases (11.0%); lower back pain: 53 cases (11.0%); synovitis, meniscus, and cartilage injuries: 37 cases (8.0%); and fractures: 29 cases (6.0%). The TL injury rate for muscle injuries was higher compared to ligament injuries in the ankle.
Owoeye et al., (2020) [46]	N = 809 youth basketball players	Sprains, Knee, Ankle, Fingers, and Hip	Most common injury type was as follows: sprains (62.5% of all injuries). Most frequently affected body parts included the following: knee: 13 cases (40.6%) and ankle: 7 cases (21.9%). Least affected areas included the following: forearm, wrist, fingers, hip, thigh, and leg (1 case each, 3.1%). Injury distribution on the court included the following: offensive half: 13 injuries (41%); defensive half: 8 injuries (25%); and key area: 11 injuries (34%).
Patel et al., (2020) [27]	Male NBA basketball players	Ligament Strain and Rupture	A total of 79 adductor injuries were recorded among 65 NBA players (2009–2019). Injury types included the following: strains: 72 cases (91%) and ruptures: 7 cases (9%). Injury frequency included the following: 7.9 ± 5.4 injuries per season (median: 7). Injury incidence was as follows: 0.27 injuries per 1000 AE during the study period. Highest number of injuries was recorded in 2019 (20 cases; 0.67 per 1000 AE). Lowest number of injuries was recorded in 2010 (1 case; 0.03 per 1000 AE). There was a significant difference between strains and ruptures (*p* = 0.003).
Mateos Conde et al., (2022) [47]	N = 223 basketball players (120 professional and 103 amateur)	Ankle Sprains, Thigh Muscle Injuries, and Knee	Total injury rate was as follows: 11.6 injuries per 1000 h of sports activity; training: 9.6 injuries per 1000 h; and competition: 47.3 injuries per 1000 h. Injury distribution included the following: ankle sprains: 50%; thigh muscle injuries: 12.2%; and patellar tendinitis: 7.4%.
Minghelli, et al., (2022) [48]	N = 126 basketball players from southern Portugal	Ankle Sprains	Injury incidence was as follows: 2.72 injuries per 1000 h of training. Most common injury type was as follows: sprains (43.8%). Most affected body part was as follows: ankle (40.1%). Main injury mechanism was as follows: contact with another player (19.4%).
Tummala, et al., (2022) [49]	N = 271 NBA players	Knee	Injury incidence per minutes played per game (per 1000 AE) was as follows: 30 min: 3.86; 20.0–29.9 min: 3.03; and 10.0–19.9 min: 1.82. A total of 740 knee injuries were reported, including 212 structural injuries affecting 21% of players.
Abdollahi and Sheikhhoseini (2022) [50]	Male basketball players (Professional Super League and First League)	Ankle, Lower back/Pelvis, Knee, Joints/Fingers, and Lower leg/Calf	Total injuries was as follows: 628 (6.07 injuries per 1000 h). Injury distribution included the following: ankle: 116 cases (26.9%), including 20.3% due to overuse; lower back/pelvis: 67 acute injuries (15.5%) and 23 due to overuse (11.6%); knee: 62 acute injuries (15.7%) and 31 due to overuse (14.3%); joints/fingers: 85 acute injuries (13.4%) and 8 due to overuse (4.0%); and lower leg/calf: 35 acute injuries (8.1%) and 28 due to overuse (14.2%).
Tosarelli et al., (2024) [51]	Male basketball players (Professional Leagues)	Knee (ACL Injuries)	Injuries occurred during offense (69%) and defense (31%) included the following: indirect injuries: 58% and non-contact injuries: 39%.

**Table 2:** Prevalence of sport injuries in volleyball.

First Author and Year	Participants	Localization	Key Findings
Bere et al., (2015) [31]	Male and female volleyball players	Ankle, Knee, Lower back, and Fingers (thumb)	Ankle injuries: 25.9%. Knee injuries: 15.2%. Finger/thumb injuries: 10.7%. Lower back injuries: 8.9%. Injury prevalence was higher among middle blockers and lower among liberos.
Huang et al., (2015) [52]	South Korean female volleyball players—2014 Asian Games	Ankle, Knee, Foot, Fingers, and Shoulder	Injuries that occurred during matches were most commonly lower extremity injuries, particularly affecting the ankles, knees, and feet, during jumps and landings. The most frequent injuries were knee injuries (33.3%) and lower back injuries (23.8%), followed by ankle injuries (16%), finger injuries (16%), and shoulder injuries (12%).
Pastor et al., (2015) [53]	N = 34 professional volleyball players	Back	Total injuries recorded: 186. Prevalence of acute injuries: 1.94 per player. Most common injuries: minor spinal injuries. Injury rate per season: second season: 1.92 injuries per player; first season: 3.25 injuries per player.
Ciesla et al., (2015) [54]	N = 90 Polish female volleyball players	Ankle, Knee and Lower Leg Muscles	Over 87% of participants sustained at least one injury. The total number of injuries recorded was 362. The most common injuries were ankle injuries (46 cases), knee and lower leg muscle injuries (30 cases), and finger and shoulder injuries (30 cases). More than half of the injuries (57%) occurred two or three times. The main causes of injuries were exhaustion, lack of rest, and inadequately healed previous injuries.
Reeser et al., (2015) [55]	High school and college female volleyball players	Ankle, Knee, and Shoulder	During the four-year study, the injury rate in college volleyball was 3.3 times higher than in high school. The most common injuries included ligament sprains, muscle strains, fractures, and concussions. Knee injuries were significantly more frequent among college players, particularly overuse injuries (38.1%), while acute ACL injuries (9.3%) were more prevalent among high school players. The most common diagnosis was patellar tendinosis (19.3%). Shoulder injuries were more common in college athletes, mainly due to spiking (37.8% in college, 39.1% in high school).
Yang et al., (2016) [56]	N = 94 male and female volleyball players	Knee, Lower back, Elbow, and Ankle	Frequent injuries include the knee (25.9%), lower back (13.3%), and ankle (8.4%). The most affected tissues are joints (41.6%) and muscles (30.7%). Common treatment approaches consist of acupuncture (40.4%), Chuna therapy (16.0%), and physical therapy (15.2%).
Cuñado-González et al., (2019) [57]	N = 490 elite Spanish volleyball players	Ankle, Knee, and Shoulder	The study was carried out over a single season, during which 71.2% of the players (490) completed an injury questionnaire. The overall injury prevalence was 66.9%, with an average of 0.94 injuries per player. The anatomical regions most affected were the ankle, knee, and shoulder, while the most common types of injuries were strains, sprains, and tendinopathies. Injuries most frequently occurred during blocking and attacking movements. A total of 90.3% of players participated in preventive programs.
Lesman et al., (2020) [58]	N = 98 women and N = 301 men	Musculoskeletal Injuries	The study was conducted over two league seasons on a weekly basis using a specialized survey. A total of 45% of all players experienced injuries and musculoskeletal trauma, including 56% of men and 26% of women. Injury incidence during matches was as follows: 17.3–33.8 injuries per 1000 h of play. Almost 50% of musculoskeletal issues were reported during training. Acute injuries were as follows: knee and ankle joints, shoulder, spine, and abdomen.
Wasser et al., (2021) [59]	N = 276 volleyball players aged 13–18	Musculoskeletal Injuries	Injuries were more common in advanced volleyball players (73%) than in beginners (62%). Higher training volume experienced among advanced players (490 h) compared to beginners (302.3 h). Beginners and intermediate-level players had a higher likelihood of elbow injuries compared to advanced players. A total of 67% of volleyball players experienced one or more injuries. Most common injuries were as follows: ankle: 40.6%; fingers: 36.6%; knee: 21.2%; and shoulder: 15.5%
Baugh et al., (2018) [60]	N = 18,844 NCAA men’s and women’s volleyball athletes	Ankle and Knee	A total of 593 injuries were recorded (83 in men and 510 in women), with a higher injury rate in women (7.07 per 1000 AEs compared to 4.69 in men). Time-loss injury rates were 1.75 for men and 2.62 for women. Ankle injuries were the most common among time-loss injuries, while the knee was the most frequently injured body part among non-time-loss injuries. Women had a higher rate of overuse injuries (IRR, 3.47).
Skazalski et al., (2024) [61]	N = 75 volleyball players	Knee, Back, and Shoulder Injuries	The study was conducted using a weekly questionnaire from the Oslo Sports Trauma Research Center. The majority of volleyball players (58%) experienced knee problems. Lower back and shoulder injuries affected 27% of players. Almost all participants reported at least one match with reduced sports performance due to injuries.
Obama et al., (2024) [62]	Young volleyball players in the USA	Epidemiology, Mechanisms, and Diagnoses of Upper Extremity Injuries	A total of 131,624 injuries were recorded in emergency departments from January 1, 2012, to December 31, 2012. Volleyball players were as follows: 77.6% female and 22.4% male. Most common injuries were as follows: sprains: 42.6% and fractures: 19.5%. Fingers were the most affected: 57.4%. Female volleyball players had higher rates of the following: contusions (16% vs. 9.9%) and strains/sprains (46.1% vs. 30.4%). Young athletes were the most exposed to upper extremity injuries, particularly in the fingers, wrists, and shoulders.
Timoteo et al., (2021) [63]	N = 14 elite volleyball players	Traumatic Injuries	Out of a total of 64 injuries, 53 (83%) were due to functional overuse (11.59 injuries per 1000 h), while 11 (17%) were traumatic injuries (2.41 injuries per 1000 h). Injury incidence was higher during the preseason (*p* = 0.003), with greater weekly workload (*p* = 0.008) and a higher acute:chronic workload ratio (ACWR) (*p* < 0.001) compared to the competitive season. Healthy players had a lower ACWR (*p* = 0.002) compared to injured players. Players who sustained injuries (both from overuse and trauma) had higher ACWR and lower Total Quality Recovery (TQR) scores than non-injured players.
Jandhyala et al., (2024) [13]	Volleyball players and injuries occurring from 2013 to 2022	Sprains, Strains, and Fractures	Total number of injuries was as follows: 347,395 (2013–2022). The most commonly injured body parts were lower extremities (40.8%) and upper extremities (34.3%), while head or neck injuries accounted for 17.1% of cases. The most common diagnoses were sprains or strains (43.6%), followed by fractures (10.3%) and bruises or abrasions (10.6%).
Deddy et.al., (2024) [64]	Volleyball players aged 17–24	Sprains, Strains, and Fractures	The most common injuries among volleyball players were minor injuries (85.33%), such as bruises and cramps, followed by moderate injuries (12.36%), including joint injuries and strains. Severe injuries (2.32%) included fractures, as well as sprains and strains.
Biese et al., (2024) [65]	N = 150 female volleyball players	Shoulder and Knee	Shoulder and knee pain in high school female volleyball players assessed through an online survey. Main cause of pain: overuse: reported by more than 60% of participants. Medical rehabilitation: not sought in 66% of cases for shoulder pain; not sought in 60% of cases for knee pain. Medical professional recommendation included the following: Only 11% of athletes rested from sports based on a doctor’s advice.
Mizoguchi et al., (2024) [66]	N = 82 high school female volleyball players	Knee and Lower Back	The study included 82 female volleyball players, with a knee pain prevalence of 19.5%. Significant factors associated with knee pain included pre-existing lower back pain (OR 4.64; *p* = 0.019) and reduced flexibility (OR 1.37; *p* = 0.037).

**Table 3:** Prevalence of sport injuries in handball.

First Author and Year	Participants	Localization	Key Findings
Bere et al., (2015) [63]	N = 384 during the 24th Men’s Handball World Championship 2015	Ankle, Thigh, Knee, Head, and Face	From 96.7% of the submitted data for all players, 27.1% of players sustained injuries, with a total incidence of 104.5 injuries per 1000 player-hours. Line players were the most frequently injured. The most common injuries occurred to the ankle, followed by the thigh, and then the knee, and finally head and face injuries.
Møller et al., (2017) [67]	N = 679 youth handball players aged 14–18 years	Shoulders	An increase in workload by more than 60% led to a higher rate of shoulder injuries (HR 1.91; 95% CI 1.00 to 3.70, *p* = 0.05). An increase in workload between 20% and 60% increased the risk of injuries in players with reduced external rotational strength (HR 4.0; 95% CI 1.1 to 15.2, *p* = 0.04) or scapular dyskinesis (HR 4.8; 95% CI 1.3 to 18.3, *p* = 0.02).
Giroto et al., (2017) [68]	N = 339 Brazilian elite handball players	Ankles, Knees, Shoulders, Muscle Injuries, and Tendinopathy	The injury incidence rate during training was 3.7 per 1000 h, while, during matches, it was 20.3 per 1000 matches. Traumatic injuries was as follows: ankle 19.4%, knee 13.5%, and muscle injuries 27.1%. Overuse injuries was as follows: shoulder 44.0%, knee 26.7%, and tendinopathy 91.8%.
Aasheim et al., (2018) [69]	N = 145 junior handball players	Knees, Shoulders, and Lower back	The average prevalence of all injury-related problems caused by overuse was 39% (95% CI 29% to 49%). The average prevalence of problems that led to reduced or inability to participate was 15% (95% CI 13% to 17%). During the 10-month study period, the cumulative incidence of overuse injury-related problems was 91% (133 players). The most common problems, with an average prevalence of 17% (95% CI 16% to 19%), were recorded in the shoulder, while knee problems had the highest relative workload impact. A total of 91% of players reported at least one problem in the examined areas. Lower back pain was the third most frequently recorded injury.
Rafnsson et al., (2019) [70]	N = 185 Icelandic elite handball players	Knee, Ankle, Foot/Toes, Lower Back/Pelvis, and Shoulder	The injury incidence rate during training was 1.1 per 1000 h, while, during matches, it was 15.0 per 1000 matches. Traumatic injuries (53 cases, 62%): Knee (26%), ankle (19%), and foot/toes (17%). Overuse injuries (33 cases, 38%): Lower back/pelvis (39%), shoulder (21%), and knee (21%).
Mónaco et al., (2019) [71]	N = 164 (youth = 133 and adults = 31)	Knee, Cartilage, Ankle, Muscles, Thigh, Head, and Apophysitis	The injury incidence rate among young handball players was 6.0 per 1000 h, with 14.9 per 1000 h during matches and 3.7 per 1000 h during training, with a total of 142 injuries recorded. The injury incidence rate among adult handball players was 6.5 per 1000 h, with 22.2 per 1000 h during matches and 3.0 per 1000 h during training, with a total of 48 injuries recorded. The study showed that adult handball players sustained more injuries to the ankle (*p* = 0.03), muscles (*p* = 0.02), thigh (*p* = 0.05), and head (*p* = 0.05) compared to young players. Backcourt and center players had a higher prevalence of knee (*p* = 0.05) and cartilage (*p* = 0.05) injuries. A higher incidence of apophysitis was observed in young handball players.
Åman et al., (2019) [72]	Swedish handball players during the period from 2006 to 2015	Knee and Head	The knee is the most frequently injured body part among handball players of all ages and both genders. Knee injuries have the highest incidence of long-term consequences, including permanent medical impairment. Head injuries and concussions are less common but still occur in both male and female players. Preventive programs focused on enhancing knee stability are essential for reducing the risk of severe injuries in handball.
Asai et al., (2020) [73]	N = 7110 (M = 3780 and F = 3330) Young handball players	Ankle, Foot, Knee, Wrist, Hand, Head, and Face	The injury rate per 1000 match hours was 26.5, meaning one injury occurred every 3.2 matches. Injuries were more common among boys than girls. There was no confirmed correlation between injury frequency and player position: lower extremities: 43.2%; upper extremities: 20.7%; head and face injuries: 31.4%; ankle and foot injuries: 24.3%; knee injuries: 13.6%; wrist and hand injuries: 13%.
Mashimo et al., (2021) [3]	N = 1017 (F = 555 and M = 462) Japanese university handball players	Ankles, Knees, Shoulders, and Lower back	The incidence rate over the one-year study period was 0.59 injuries per player per year (95% CI: 0.56–0.62). A total of 469 players (46.1%) out of 1017 reported at least one injury. Comparing gender differences, 185 out of 462 male players (40%) sustained an injury, while 284 out of 555 female players (51.2%) were injured. Women were 1.5 times more likely to experience an injury than men. Regarding player positions, line players were also 1.5 times more likely to sustain an injury compared to wing players. The most common traumatic injuries were ankle injuries (33.3%), followed by knee injuries (23.6%) and shoulder injuries (12.6%). Overuse injuries were most frequently reported in the lower back (26.0%), followed by the knee (15.7%) and shoulder (15.0%).
Raya-González et al., (2021) [74]	N = 27 professional Spanish handball players	Ankle, Knee, Ligaments, and Thigh	The study monitored injuries over two seasons between an experimental and a control group. The injury incidence rate during training was 2.27 per 1000 h for the control group and 1.75 per 1000 h for the experimental group, while the injury incidence rate during matches was 26.8 per 1000 h for the control group and 23.0 per 1000 h for the experimental group. No significant differences were recorded between the control and experimental seasons regarding injury location (ankle, knee, and thigh) or injury type (ligament sprains, muscle injuries, and tendon injuries). The injury incidence per 1000 h of exposure was as follows: ankle injuries (0.48 control/0.40 experimental), knee injuries (0.66 control/0.60 experimental), ligament injuries (0.56 control/0.49 experimental), and thigh injuries (0.15 control/0.16 experimental).
Roh et al., (2021) [75]	N = 188 (96 M; 92 F) elite South Korean handball players	Lower Extremities	The average annual injury rate per athlete was 4.08. Female athletes had a higher injury incidence rate of 6.21 per 1000 h, compared to 4.39 per 1000 h for male athletes. The most common injuries occurred in the lower extremities, accounting for 50.2% of all injuries. Injured body areas varied based on player position (*p* < 0.001), while injury severity differed by gender (*p* < 0.001), with male athletes experiencing a longer recovery period. Pain intensity and recovery time significantly differed between genders (*p* < 0.001), with ligament injuries associated with the highest pain levels and longest recovery time. For every one unit increase in pain level, the recovery period increased by 1.59 days.
Barič et al., (2021) [76]	Players from five senior Slovenian handball leagues during the 2010/2011 season.	Ankle, Knee, and Shoulder	A total of 92 injuries were reported, affecting 45% of all players, with an average of 0.58 injuries per player (57 in women and 35 in men). The majority of injuries (33.3%) resulted from contact situations. The injury incidence rate for women was 27.7 injuries per 1000 match hours and 0.97 injuries per 1000 training hours, while, for men, it was 10.6 injuries per 1000 match hours and 0.5 injuries per 1000 training hours. Left and right backs were the most frequently injured positions (41%). The most common injury type was sprain (55%), and 29% of all injuries were classified as severe. A total of 50% of shoulder injuries and over 70% of knee injuries were categorized as severe. A total of 62% of injuries affected the lower extremities, with the ankle accounting for 35%. A total of 36% of players reported overuse injuries and syndromes affecting the shoulder and ankle.
Karlsson et al., (2021) [77]	N = 45 (23 F/22 M) adolescent elite handball players	Lower Extremities	A total of 64% of athletes reported at least one injury during the year, while 29% reported more than two new injuries. The prevalence of significant seasonal injuries was 22.2%, with 39% in female athletes and 17% in male athletes.
Mashimo et al., (2021) [78]	N = 2377 young Japanese handball players	Knee, Fractures, and Lower Leg	The overall injury prevalence during the one-season study period was 46.7%. Backcourt players sustained at least one injury more frequently than players in other positions. Traumatic injuries showed a higher percentage of ligament ruptures among backcourt players, while fractures were more common among line players (*p* = 0.011). Overuse injuries were most frequently observed in wing players and backcourt players, with the lower leg and knee being the most commonly affected areas (*p* = 0.047).
Raya-González et al., (2022) [79]	N = 68 professional handball players	Knee, Muscles, and Tendons	The highest injury burden by position included the following: full-backs: 60.65 days of absence per 1000 h (RR from 0.12 to 7.75; *p* < 0.05); wingers: 54.29 days of absence per 1000 h (RR from 0.09 to 4.91; *p* < 0.05); with a significantly lower burden in: goalkeepers: 12.19 days of absence per 1000 h; and pivots: 13.10 days of absence per 1000 h. Injury incidence by position was as follows: no significant differences in injury incidence were found among positions (RR from 0.43 to 2.47; *p* > 0.05). Most common types and locations of injuries included the following: Muscle/tendon injuries and sprains had the highest incidence and burden.Knee injuries were the most common across all positions. Full-backs are the most at risk in terms of injury incidence and burden, with knee, muscle, and tendon injuries being the most frequent.
Martínez-Aranda et al., (2024) [80]	N = 68 elite male handball players from Spain	Anterior Talofibular Ligament and Anterior Cruciate Ligament	The overall incidence of ligament injuries was 0.89 per 1000 h of exposure. Lower extremity injuries were significantly more common than upper extremity injuries (0.81 vs. 0.08 per 1000 h; *p* < 0.001). The anterior talofibular ligament of the ankle had the highest incidence (0.57 per 1000 h), while the anterior cruciate ligament had the highest injury burden (24.08 days of absence per 1000 h). A higher incidence and injury burden were observed during matches compared to training sessions. A total of 79.63% of injuries were minor or moderate, while 46.29% were reinjuries. Specific programs for strengthening, balance, and injury prevention are recommended.
Resch et al., (2025) [81]	N = 5320 professional German handball players from the 1st and 2nd leagues followed over 7 seasons	Knee (ACL Ligament—Anterior Cruciate Ligament)	Out of a total of 84 recorded injuries (38 (45%) in the first league and 46 (55%) in the second league), 63 players (75%) completed the questionnaire.The incidence rate of ACL injuries during matches and training was 0.044 per 1000 h. In the first league, there were 8.3 injuries with an incidence rate of 0.064 per 1000 h per season, while, in the second league, there were 6.6 injuries with an incidence rate of 0.031 per 1000 h. The most common situation for injuries in both leagues was landing (38.1%), followed by a change in direction (17.3%) and stopping (12.9%). In 46.3% of cases, athletes experienced a re-injury (rupture) of the ACL ligaments.

## Supplementary Materials

The following supporting information can be downloaded at https://www.mdpi.com/article/10.3390/life15040529/s1, Table S1: Newcastle-Ottawa Quality Assessment Form for Cohort Studies Basketball; Table S2: Newcastle-Ottawa Quality Assessment Form for Cohort Studies Volleyball; Table S3: Newcastle-Ottawa Quality Assessment Form for Cohort Studies Handball.
