# Supplementary material for: Sports Injuries in Basketball, Handball, and Volleyball Players: Systematic Review
Source: Life (Basel). 2025 Mar 24;15(4):529. doi: 10.3390/life15040529 (PMC12028468; doi:10.3390/life15040529)
Supplement: Supplementary file 1 [file life-15-00529-s001.zip › life-3502898-supplementary.pdf]

Table S1. - Newcastle-Ottawa Quality Assessment Form for Cohort Studies **Basketball**

Author/Year	Selection	Comparability	Outcome	Total	Quality
Leppänen et al. (2015)	• • • •	• •	• • •	8	Good
Moreira et al. (2016)	• • • •	•	• •	7	Good
Minhas et al. (2016)	• • •	•	• • •	7	Good
Riva et al. (2016)	• • •	• •	• •	7	Good
Pasanen et al. (2017)	• • • •	• •	• • •	8	Good
Garbenytė-Apolinskienė et al. (2019)	• • •	•	• •	6	Fair
Anderson et al. (2019)	• • • •	• •	• • •	8	Good
Rodas et al. (2019)	• • •	• •	• •	7	Good
Owoeye et al. (2020)	• • • •	• •	• • •	8	Good
Patel et al. (2020)	• • •	•	• •	6	Fair
Mateos Conde et al. (2022)	• • • •	• •	• • •	8	Good
Minghelli et al. (2022)	• • •	•	• •	6	Fair
Tummala et al. (2022)	• • • •	• •	• • •	8	Good
Abdollahi & Sheikhhoseini (2022)	• • •	• •	• •	7	Good
Tosarelli et al. (2024)	• • • •	•	• •	7	Good

Table S2. - Newcastle-Ottawa Quality Assessment Form for Cohort Studies **Volleyball**

Author/Year	Selection	Comparability	Outcome	Total	Quality
Bere et al. (2015)	• • • •	• •	• •	8	Good
Huang et al. (2015)	• • •	•	• •	6	Fair
Pastor et al. (2015)	• • • •	• •	• •	8	Good
Ciesla et al. (2015)	• • •	•	• •	6	Fair
Reeser et al. (2015)	• • • •	• •	• • •	8	Good
Yang et al. (2016)	• • •	•	• •	6	Fair
Cuñado-González et al. (2019)	• • • •	• •	• • •	8	Good
Lesman et al. (2020)	• • •	• •	• •	7	Good
Wasser et al. (2021)	• • •	•	• •	6	Fair
Baugh et al. (2018)	• • • •	• •	• • •	8	Good
Skazalski et al. (2024)	• • •	•	• •	6	Fair
Obama et al. (2024)	• • • •	• •	• • •	8	Good
Timoteo et al. (2021)	• • • •	• •	• • •	8	Good
Jandhyala et al. (2024)	• • •	•	• •	6	Fair
Deddy et al. (2024)	• • • •	• •	• • •	8	Good
Biese et al. (2024)	• • •	•	• •	6	Fair
Mizoguchi et al. (2024)	• • • •	• •	• • •	8	Good

Table S3. - Newcastle-Ottawa Quality Assessment Form for Cohort Studies **Handball**

Author/Year	Selection	Comparability	Outcome	Total	Quality
Bere et al. (2015)	••••	••	••	8	Good
Møller et al. (2017)	•••	••	••	7	Good
Giroto et al. (2017)	••••	•	••	7	Good
Aasheim et al. (2018)	••••	••	•••	8	Good
Rafnsson et al. (2019)	•••	•	••	6	Fair
Mónaco et al. (2019)	••••	••	•••	8	Good
Åman et al. (2019)	•••	••	••	7	Good
Asai et al. (2020)	••••	••	•••	8	Good
Mashimo et al. (2021)	•••	•	••	6	Fair
Raya-González et al. (2021)	••••	••	•••	8	Good
Roh et al. (2021)	•••	••	••	7	Good
Barič et al. (2021)	•••	••	••	7	Good
Karlsson et al. (2021)	••••	•	••	7	Good
Mashimo et al. (2021)	•••	•	••	6	Fair
Raya-González et al. (2022)	••••	••	•••	8	Good
Martínez-Aranda et al. (2024)	•••	•	••	6	Fair
Resch et al. (2025)	••••	••	•••	8	Good

Legend:

- **Selection:**
  - Representativeness of the exposed cohort;
  - Selection of the non-exposed cohort;
  - Ascertainment of exposure;
  - Demonstration that outcome of interest was not present at start of study
- **Comparability:**
  - Comparability of cohorts on the basis of design or analysis (adjusted for age);
  - Comparability of cohorts on the basis of design or analysis (adjusted for any other factor)
- **Outcome:**
  - Assessment of outcome;
  - Was follow-up long enough for outcomes to occur;
  - Adequacy of follow-up of cohorts
- **Quality Ratings:** 0-3 (Poor), 4-6 (Fair), 7-9 (Good)
